# Supplementary material for: Understanding key mechanisms of successfully leading integrated team-based services in health and social care: protocol for a realist synthesis
Source: BMJ Open. 2020 Jul 9;10(7):e038591. doi: 10.1136/bmjopen-2020-038591 (PMC7351270; doi:10.1136/bmjopen-2020-038591)
Supplement: Author's manuscript [file bmjopen-2020-038591.draft_revisions.pdf]

BMJ Open is committed to open peer review. As part of this commitment we make the peer review history of every article we publish publicly available.

When an article is published we post the peer reviewers' comments and the authors' responses online. We also post the versions of the paper that were used during peer review. These are the versions that the peer review comments apply to.

The versions of the paper that follow are the versions that were submitted during the peer review process. They are not the versions of record or the final published versions. They should not be cited or distributed as the published version of this manuscript.

BMJ Open is an open access journal and the full, final, typeset and author-corrected version of record of the manuscript is available on our site with no access controls, subscription charges or pay-per-view fees (http://bmjopen.bmj.com).

If you have any questions on BMJ Open's open peer review process please email info.bmjopen@bmj.com

BMJ Open

Understanding key mechanisms of successfully leading integrated team-based services in health and social care: Protocol for a realist synthesis

Journal:	*BMJ Open*
Manuscript ID	bmjopen-2020-038591
Article Type:	Protocol
Date Submitted by the Author:	17-Mar-2020
Complete List of Authors:	Harris, Ruth; King\'s College London,<br>Fletcher, Simon; Kingston and St Georges University London, Health, Social Care and Education<br>Sims, Sarah; King\'s College London, Florence Nightingale Faculty of Nursing and Midwifery<br>Ross CBE, Fiona; Kingston University and St. George's, University of London, Faculty of Health, Social Care and Education<br>Brearley, Sally; King's College London, Florence Nightingale Faculty of Nursing and Midwifery<br>Manthorpe, Jill; King's College London, Health & Social Care Workforce Research Unit
Keywords:	Organisation of health services < HEALTH SERVICES ADMINISTRATION & MANAGEMENT, Human resource management < HEALTH SERVICES ADMINISTRATION & MANAGEMENT, Health policy < HEALTH SERVICES ADMINISTRATION & MANAGEMENT

SCHOLARONE™
Manuscripts

I, the Submitting Author has the right to grant and does grant on behalf of all authors of the Work (as defined in the below author licence), an exclusive licence and/or a non-exclusive licence for contributions from authors who are: i) UK Crown employees; ii) where BMJ has agreed a CC-BY licence shall apply, and/or iii) in accordance with the terms applicable for US Federal Government officers or employees acting as part of their official duties; on a worldwide, perpetual, irrevocable, royalty-free basis to BMJ Publishing Group Ltd ("BMJ") its licensees and where the relevant Journal is co-owned by BMJ to the co-owners of the Journal, to publish the Work in this journal and any other BMJ products and to exploit all rights, as set out in our licence.

The Submitting Author accepts and understands that any supply made under these terms is made by BMJ to the Submitting Author unless you are acting as an employee on behalf of your employer or a postgraduate student of an affiliated institution which is paying any applicable article publishing charge ("APC") for Open Access articles. Where the Submitting Author wishes to make the Work available on an Open Access basis (and intends to pay the relevant APC), the terms of reuse of such Open Access shall be governed by a Creative Commons licence – details of these licences and which Creative Commons licence will apply to this Work are set out in our licence referred to above.

Other than as permitted in any relevant BMJ Author's Self Archiving Policies, I confirm this Work has not been accepted for publication elsewhere, is not being considered for publication elsewhere and does not duplicate material already published. I confirm all authors consent to publication of this Work and authorise the granting of this licence.

For peer review only

Understanding key mechanisms of successfully leading integrated team-based services in health and social care: Protocol for a realist synthesis.

Corresponding author:
Ruth Harris
Florence Nightingale Faculty of Nursing, Midwifery and Palliative Care, King's College London,
James Clerk Maxwell Building, 57 Waterloo Road, London, SE1 8WA, UK
Email: ruth.harris@kcl.ac.uk
Tel: 02078483708
ORCID ID: 0000-0002-4377-5063

Simon Fletcher
Faculty of Health, Social Care and Education,
Kingston University and St George's, University of London, London, UK

Sarah Sims
Florence Nightingale Faculty of Nursing, Midwifery and Palliative Care,
King's College London, London, UK

Fiona Ross CBE
Faculty of Health, Social Care and Education,
Kingston University and St George's, University of London, London, UK

Sally Brearley
Faculty of Health, Social Care and Education,
Kingston University and St George's, University of London, London, UK

Jill Manthorpe
NIHR Policy Research Unit on Health and Social Care Workforce,
King's College London, London, UK
ORCID ID: 0000-0001-9006-1410

Word count: 3269 excluding title page, abstract, references, figures and tables.

For peer review only

Abstract

Introduction: As systems of health and social care in England move towards more integrated and collaborative models, their professional teams are thought to require distinctive leadership in order to improve outcomes for patients and service users. The turbulence which cross organisational working provokes may require those in leadership positions to negotiate any resulting tensions, remaining as close to the shared objectives of service improvement as possible. Research indicates that, firstly, lack of clarity in leadership and conflict in leadership have negative implications for team performance, and secondly, that there is an evidence gap in what is known about how leaders work across organisational boundaries of health and social care in integrated systems. This suggests a need to explore the complexity of the leadership of integrated teams in greater depth.

Methods and Analysis: This study utilises a realist synthesis approach to explore the existing literature on leadership of integrated teams. This process is being complemented by ongoing stakeholder and advisory group consultation. The development of realist programme theories which point to optimal leadership characteristics has the advantage of iterative engagement with a range of literature sources and provides a central, practically applicable picture of the evidence base.

Ethics and Dissemination: Our study does not require ethics committee approvals. This research will contribute to building an in-depth understanding of what aspects of leadership of integrated team-based services work, for whom and in what circumstances and clarify activities contributing to leadership roles and responsibilities. In identifying the development needs of the leaders of integrated team-based health and social care services we will inform professional development and provide recommendations to ongoing decision making about optimal organisational and inter-organisational structures and processes that support effective leadership in integrated health and social care systems.

Systematic review registration: PROSPERO Number CRD42018119291

Keywords: Integrated care, leadership, realist synthesis, health and social care

For peer review only - http://bmjopen.bmj.com/site/about/guidelines.xhtml

Strengths and limitations of this study

- The realist review will articulate the preliminary theories and assumptions about leadership of complex integrated health and social care teams and how it is expected to work.
- The review will test and refine these theories using existing empirical evidence and consultation with an expert stakeholder group to provide current understanding of what works (and does not work), for whom, in what circumstances and how.
- Consultation with stakeholders with a range of expertise and experience will enable the review to be grounded in the reality of health and social care delivery and address practice and policy challenges.
- The landscape of integrated care in health and social care is rapidly developing and thus the context in which leaders work will change accordingly and so the recommendations of the review may need further consideration.

For peer review only

For peer review only - http://bmjopen.bmj.com/site/about/guidelines.xhtml

Background

The Long Term Plan for the UK National Health Service (NHS) and its predecessor strategy, The NHS Five Year Forward View, set out a clear direction for how health and social care services in England must develop to deliver high quality care and treatment in the context of changing patient/user need, increased service delivery pressures and restrained budgets.[1,2] The NHS already works across a wide range of providers and within several sectors and its interdependence between health and social care will certainly increase with new partnerships with local communities, local authorities, and employers being implemented.[2] These new integrated care systems aim to bring together organisations to plan and oversee the implementation of improvements in health and care. It has been argued that current and future integrated care systems must address a range of development needs if they are to be successful, with the development of integrated leaders being vital to this shift.[3]

Leadership is a complex concept. While definitions vary, there is a consensus that a leadership role encompasses the direction of group activities towards shared goals, management of on-going change and support for wider organisational vision, values and objectives.[4,5] Although there are many definitions, some of which seek to differentiate it from management,[6] effective leadership is claimed to be a central element of well-coordinated and safe care.[7-11] Where leadership in health and social care organisations is ineffective (or absent), services fail to meet their aims and patients and service users may be at risk of harm.[12]

Existing research on leadership tends to be based on the notion that leaders provide guidance and support for members of single or uni-professional teams, often based on seniority, authority and deferment.[13,14] However, as the organisation of the NHS and local authority social care commissioning in England becomes more integrated through increasing multi-party, cross-sector and interagency collaboration,[1,15] there is growing acknowledgement that leadership is becoming considerably more complex, with specific factors making effective leadership more challenging,[16,17] particularly in negotiating the intricacy and inevitable tensions of expanding integration. Clarity of leadership or conflict

For peer review only - http://bmjopen.bmj.com/site/about/guidelines.xhtml

in leadership in interprofessional teams has also been found to be highly significant predictors of team performance[18-21] yet there appears to be a tendency for professionals to focus on the support and guidance provided by their own professional leader rather than the leader of the larger interprofessional team.[18,21]

These ongoing and future changes in service delivery mean that health and social care leaders will be less often influencing just one organisation or professional group but instead will be working between several organisations across primary and secondary care, health and social care and publicly funded services, the not-for-profit sector and private businesses. Today's health and social care leaders will therefore need different skills than their predecessors to enable system leadership, building partnerships and working across organisations and sectors.[22,23] However, there is little understanding of what the mechanisms for effective leadership across integrated health and social care systems might be, the contexts that influence good leadership, or the nature of the resulting outcomes.[16] Given this gap in our knowledge and the pressing need to know what might support good leaders in this policy and practice priority area, this realist review focuses on identifying what constitutes good leadership across professional, sector and agency boundaries that seek to promote integrated team-based services across health and social care. The review will undertake an extensive search of the health and social care literature to identify the concepts/theories of leadership to examine what mechanisms work, for whom and in what circumstances. The results from this synthesis, informed by patient, user and carer perspectives, will support the ongoing development and organisation of health/social care, inform leadership development programmes and refine theoretical understandings of leadership to enable future research.

Aims and objectives

This review aims to identify and refine the programme theories of leadership of integrated team-based services in health and social care, exploring what works, for whom and in what circumstances. It will provide practical guidelines for policymakers, health and social care leaders, managers and clinicians to help them design work systems and leadership development initiatives to support effective leadership of complex multi-system services. The review started in April 2019.

For peer review only - http://bmjopen.bmj.com/site/about/guidelines.xhtml

Objectives:

1. To investigate who are the leaders of integrated team-based services and what activities contribute to their leadership roles and responsibilities.
2. To explore how leaders lead/manage integrated team-based health and social care services that span multiple organisations, agencies and sectors.
3. To develop realist programme theories that explain successful leadership of integrated team-based health and social care services iteratively through stakeholder consultation and evidence review
4. To identify the development needs of the leaders of integrated team-based health and social care services.
5. To provide recommendations about optimal organisational and inter-organisational structures and processes that support effective leadership of the integrated health and social care system.

Methods and analysis

As with all complex social interventions, it can be assumed that leadership *might* work for different stakeholders in various settings in different ways. Therefore, we will conduct a realist review,[24,25] developing and iteratively refining initial programme theories through stakeholder consultation and evidence review. This approach has been successfully used previously by the research team members.[18,26,27] Realist synthesis[28] was developed in order to apply realist methods[29] to the evaluation of evidence. It may be of particular use when exploring a concept as fluid as leadership, as the processes of theoretical reasoning which are required by the approach will enable an interrogation of systems and policy to a depth not possible with more conventional or systematic evidence reviews. Pawson (2006, pp. 73-74)[28] states that: 'There is a need for systematic review to go beyond reportage and summary of an existing state of affairs. The point after all, is to support fresh thinking to revise policy and launch it in new directions'. This suggests an inherently iterative, non-linear approach to encompass multiple literature searches and the constant refinement of the evidence-based programme theories. Appraisal of the literature should, in addition, be driven by how well it 'fits' into this process rather than being guided by predetermined quality criteria. Utilising this method also allows for the plurality of leadership strategies, the

For peer review only - http://bmjopen.bmj.com/site/about/guidelines.xhtml

success of which seem often dependant on the unique combination of specific contextual conditions and associated actions.

Our realist review will employ a range of strategies, including searches of relevant research and 'grey' literature, health and social care policy documents, and stakeholder and advisory consultation. Given the potential complexity of this review, we plan three separate stakeholder and advisory consultation events during the project to 'ground' the review in the lived experiences of leaders of integrated teams and patient/user/carer and public involvement (PPI) members of the review advisory group. We will invite 8-10 individuals with health and social care leadership experiences, realist review expertise and/or health/social care leadership policymaking experience. These individuals will meet with the study advisory group and the research team to elicit "realist theories" on the mechanisms and contexts of leadership. This process is recommended in realist evaluation,[29] as understanding what key stakeholders know about an intervention and their reasoning for or against its implementation are essential to understanding it.

The review will be conducted in three interlinked phases:

Phase 1 *– Development of initial programme theories*

In the first phase, initial programme theories, i.e. purported ideas of 'what is supposed to happen?' or 'how is it supposed to work?' will be identified and made explicit. This will be undertaken in two ways:

- By examining research, policy and grey literature about leadership of complex integrated teams. Research literature will be identified by electronic searches of databases including Medline, CINAHL, Embase, PsycINFO, Health Management Information Consortium, government and other specialist health and social care websites. The following search strategy will be used in phase 1: "Integrat*" OR "multi-team*" OR "multiteam*" OR "cross-bound*" OR "cross bound*" OR "cross-organisation*" OR "cross organisation*" OR "cross-sector*" OR "cross sector*" AND "leader*" (Limiter: English language only, where available). Grey literature relating to policy and organisational-based material will be sought by searching Google Scholar,

For peer review only - http://bmjopen.bmj.com/site/about/guidelines.xhtml

government and other specialist websites (e.g. Leadership Academy, Skills for Care, King's Fund, Advance HE, The Institute of Healthcare Management, Social Care Online). The research team will independently examine documents to identify any purported mechanisms of leadership (i.e. theories or assumptions about why/how leadership was successful/was expected to work). This will continue until no new mechanisms are identified. Discussion between the research team will generate, through consensus, a combined list of preliminary context-mechanism-outcome configurations of leadership of complex integrated teams/services to be refined throughout the review.

- By consulting with key stakeholders and advisory experts to elicit their theories and assumptions about how leadership of integrated teams works. At the first consultation event, participants will be asked to think about their own knowledge and experience of leadership. For example, health and social care leaders will be asked to comment upon their own personal experiences of leading different teams/services and discuss instances when they felt it worked particularly well or not so well. In realist evaluation, such information is useful for its insight and explanatory nature, which can help identify the contexts and mechanisms which are conducive to the outcome of an intervention.[29] It is likely that the literature identified will be broad and several initial programme theories will be identified. This first stakeholder and advisory consultation event will provide important insights into current (early 2020) priorities around leadership within integrated care that will inform the scope of the literature search and direct the research team towards more pertinent aspects of leadership of complex, integrated teams.

Phase 2 – *Retrieval, review and synthesis of evidence*

In this phase, empirical evidence will be sought and reviewed to refine the programme theories. This phase will be undertaken by two activities:

<u>Evidence review</u>

Literature searching and screening. First, empirical literature will be identified from electronic searches of databases including Medline, CINAHL, Embase, PsycINFO and Health Management Information Consortium. The following search strategy will be used in phase 2: "Integrat*" OR "multi-team*" OR "multiteam*" OR "cross-bound*" OR "cross bound*" OR

For peer review only - http://bmjopen.bmj.com/site/about/guidelines.xhtml

“cross-organisation*” OR “cross organisation*” OR “cross-sector*” OR “cross sector*” ***OR “Interorganisation*” OR “Inter-orgnisation*”*** AND “leader*” ***AND “Health”*** (Limiter: English language only, where available)***.*** Reference lists of relevant papers will be scanned and citation searches conducted in order to identify further materials. Expert advice about generating relevant search terms will be sought from the University’s Library and Information Sciences Specialists and revised as additional key words are generated. Papers and other information that satisfy any of the following criteria will be identified as potentially relevant and will be retrieved for review: describe or evaluate leadership; detail its implementation or development in various settings; address the experience of team leaders, team members, policy makers related to leadership; describe the organisational or political context of leadership; reviews of leadership. Only English language documents will be included in the review of the literature. In line with realist methodology,[30] we will not have specific predetermined inclusion and exclusion criteria based upon research method or quality, but we will report areas of general weakness in evidence and individual study weakness where appropriate. Documents will be selected as relevant based on what they can contribute to theory development and/or refinement. The abstracts of all papers identified by searches will be screened for suitability.

Extraction of key information. All potentially relevant papers will be retrieved and assessed by a member of the research team using a structured data extraction form. It is envisaged that the following information will be recorded for each potentially relevant paper: literature item details – whether descriptive, evaluative or a review paper; health and social care service areas in which leadership is situated; description of leadership activities; any reported outcomes in relation to leadership activities, enabling or inhibiting contexts linked to leadership; clarification and explanation about context-mechanism-outcome configurations. Each of the data extraction forms will be independently examined by at least two members of the research team for inclusion. Data or information from each of the included materials will be analysed thematically to provide a comprehensive description of the purported mechanisms of team leadership. Contexts that appear to trigger or inhibit the mechanisms will be identified and outcomes for health and social care staff, teams, organisations and patients/users/carers/family members

For peer review only - http://bmjopen.bmj.com/site/about/guidelines.xhtml

when the mechanism is present or absent will be noted.

Analysis and synthesis. All extracted information will be analysed and synthesised to identify the relationships between identified mechanisms, contexts and outcomes. This process will draw on the realist review work of Rycroft-Malone et al. (2012)[31] and Wong et al. (2016; 2017)[32,33] which build on Pawson's (2006)[28] earlier work on realist enquiry. In doing so, we will undertake the following: organisation of extracted information into evidence tables representing different bodies of literature; identification of themes across evidence tables in relation to emerging patterns in between mechanisms, contexts and outcomes; and linking the emerging patterns to develop hypotheses. Analysis and synthesis will occur iteratively and are likely to run in parallel, with analysis informing syntheses. We will identify prominent recurrent patterns of context and outcome configurations and seek to explain how these occurred.

<u>Stakeholder and advisory consultation</u>

At the second event, participants will be asked to provide advice to the research team with the volume of data generated from the searches. If available theories are limited within the literature, the consultation event could generate additional theories that were not previously identified. If the number of theories is unwieldy, the stakeholder event will enable participants to advise the research team on which should be selected for further investigation.

Phase 3 *– Testing and refining of the initial programme theory*

In this phase planned for 2020, further refinement and testing of the programme theories will be undertaken by juxtaposing, adjudicating, reconciling, consolidating and situating the evidence analysed in phase 2.[28] This will generate a revised programme theory. This final phase will consist of the following activities:

- The research team will interrogate each explanatory inference identified in Phase 2 to elicit how each primary study supports, weakens, modifies and refocuses the initial programme theories and how particular mechanisms produce outcomes, within specific contexts. Comparisons between contexts and different types of health and

For peer review only - http://bmjopen.bmj.com/site/about/guidelines.xhtml

For peer review only

social care services will be sought to test the refined programme theories and draw out patterns of demi-regularity. Furthermore, the refined programme theories will be tested against substantive theory on leadership of complex systems.

- At the final consultation event (mid 2020), interpretations from Phase 3 of the review will be tested through the experiences and expertise of the stakeholder/advisory group. Recommendations will be sought about what leadership mechanisms may be of most benefit and what contextual factors might support their success. Furthermore, participants will be asked to consider how best to present study findings and outputs to be useful to the NHS and social care system.

Ethics and dissemination

As this is a review and synthesis of literature no formal ethical approval is needed. However, the team will apply good practices of research governance and conduct. We will follow RAMESES Guidelines[32,33] when reporting the findings from this review. It is anticipated that the final report containing synthesised review findings will identify the underlying mechanisms of leadership of integrated team-based services, and explain how these produce their effects, as well as highlight the main contextual factors that impact success or failure. Recommendations can then be made as to how employers can best target or develop integrated team leadership development for specific groups in various settings.

We will draw on the networks and expertise of the research team, advisory group and collaborators to disseminate the research outputs widely and appropriately. Key audiences are expected to include:

- Health and social care staff, managers and leaders together with clinical and human resource directors who have responsibility for the provision of health and social care in provider organisations, local authorities, voluntary and private sector as well as NHS acute, mental health and community Trusts
- Managers and directors of networks, e.g. in England these include Academic Health Sciences Networks and the NIHR Applied Research Collaborations (ARCs) who have responsibility for applied research, knowledge exchange and promoting innovation through pathways of care across a health and social care system
- Health and social care policymakers, nationally and internationally

For peer review only - http://bmjopen.bmj.com/site/about/guidelines.xhtml

For peer review only

- Health Education England, Royal Colleges and other leadership groups (e.g. NHS Leadership Academy, Skills for Care, Local Government Association, Care England, National Care Forum, King's Fund, Advance HE, The Institute of Healthcare Management, NHS Improvement), who are influential in policy concerning leadership in different contexts
- National patient/ service user and carer organisations.

Patient and public involvement

One member of the research team (SB) has a great deal of experience of patient and public involvement, for example, she is currently a Lay Member on the Interventional Procedures Advisory Committee at the National Institute for Health and Care Excellence (NICE) and has chaired many service user and carer research advisory groups at a national and local level. She has an established reputation as a co-applicant on NIHR grants and taken an active role in conduct and delivery of research, including writing for publication. As a co-applicant on this grant, she was involved in writing the protocol, developing the review questions and methods, and takes a full part in research team meetings and in particular planning the stakeholder and advisory group meetings and follow up. Three members of the public who have expertise in providing patient/service user expertise in health and social care services are part of the independent study advisory group and actively involved in the development and refinement of programme theories. Their expertise and perspectives will also be sought to develop recommendations and contribute to the study dissemination strategy.

Discussion

This realist review will enable a novel theoretical and strategically practical response to the relative lack of knowledge around the leadership of integrated health and social care services. It aligns with England's current priorities in health and social care and will provide new insights about how the leadership needs of these services are changing.

The need for integrated care teams and systems is an increasing priority in response to fragmented and un-coordinated care delivery. Ageing and diverse populations, increasing

For peer review only - http://bmjopen.bmj.com/site/about/guidelines.xhtml

inequalities, associated co-morbidities and a continual increase in long-term conditions require the collaborative interdependence of health and care providers to reduce harm and improve well-being. However, the simplicity of this statement belies the complexity of the landscape. Effective leadership is needed in order to mediate the shared objective of improving health and well-being outcomes and the altogether more difficult management of workforce interaction and the associated disruption of organisational change.

Declarations

Ethics approval and consent to participate – Not applicable

Consent for publication - Not applicable

Availability of data and material - The datasets generated and/or analysed during the current study will be available from the corresponding author on reasonable request.

Competing interests – The authors declare that they have no competing interests.

Funding - This project is funded by the National Institute for Health Research (NIHR) Health Service and Delivery Research (HS&DR) (project reference 18/01/06). The views expressed are those of the authors and not necessarily those of the NIHR or the Department of Health and Social Care.

Acknowledgements – This project was originally developed with the late Professor Scott Reeves, who passed away unexpectedly on May 3rd 2018. His original ideas and considerable expertise in interprofessional healthcare research were instrumental in the formation of this study and the research team dedicate this project to his memory.

Contributors - RH conceived the study with the late Scott Reeves and they drafted the protocol. SF, FR, SS, SB and JM contributed to reworking and refining the study objectives, design and methodology. SF and RH drafted the paper and FR, JM, SS and SB have reviewed and provided comments to improve the paper. All authors have read and approved the final version.

For peer review only - http://bmjopen.bmj.com/site/about/guidelines.xhtml

For peer review only

References

1. NHS England (2014). *The NHS Five-year forward view.* Available at: https://www.england.nhs.uk/publication/nhs-five-year-forward-view/

2. NHS England (2019). *The NHS Long Term Plan*. Available at: https://www.longtermplan.nhs.uk/

3. Ham C (2018). *A progress report on integrated care systems.* Available online at: https://www.kingsfund.org.uk/blog/2018/03/progress-report-integrated-care-systems

4. Dopson S & Annabelle M (2003). Leading Health Care Organizations. Palgrave Macmillan, Basingstoke.

5. Dopson S, Ferlie E, McGivern G, Fischer M, Ledger J, Behrens S & Wilson S (2016). *The impact of leadership and leadership development in higher education: A review of the literature and evidence.* Leadership Foundation for Higher Education, London.

6. Iliffe S & Manthorpe J (2017). Service integration through medical leadership in England's NHS. *Journal of Integrated Care* **26**, 77-86.

For peer review only - http://bmjopen.bmj.com/site/about/guidelines.xhtml

7. NHS Leadership Academy (2011). *Clinical Leadership Competency Framework.* Institute for Innovation and Improvement: Coventry.

8. Drucker P. (2002). *Managing in the Next Society.* New York: Truman Talley Books, St. Martin's Griffin.

9. NIHR (2013). *New Evidence on Management and Leadership*. National Institute for Health Research, Southampton.

10. Ahmed N, Ahmed F, Anis H, Carr P, Gauher S & Rahman F (2015). An *NHS leadership team for the future.* Reform Research Trust: London.

11. Smith T, Fowler-Davis S, Nancarrow S, Ariss S & Enderby P (2018). *Leadership in interprofessional health and social care teams: A literature review.* Leadership in Health Services. Accessed via: https://doi.org/10.1108/LHS-06-2016-0026.

12. Francis R. (2013). *Report of the Mid Staffordshire NHS Foundation Trust Public Inquiry, (Chair Robert Francis QC)*. London: The Stationery Office.

13. Reeves S, MacMillan K, van Soeren M (2010). Leadership within interprofessional health and social care teams: a socio-historical overview of some key trials and tribulations. *Journal of Nursing Management* **18**, 258-264.

14. Borkowski N (2015). *Organizational behavior in health care.* Jones & Barlett Learning: Burlington MA.

15. Ham C & Murray R (2015). *Implementing the five year forward view: aligning policy to the plan.* Available online at: www.kingsfund.org.uk/publications/implementing-nhs-five-yearforward-view

16. Fillingham D & Weir B (2014). *System leadership. Lessons and learning from AQuA's*

For peer review only

For peer review only - http://bmjopen.bmj.com/site/about/guidelines.xhtml

Integrated Care Discovery Communities. King's Fund: London.

17. Turner S, Ramsay A, Perry C, Boaden R, McKevitt C, Morris S, Pursani N, Rudd A, Tyrrell P, Wolfe C & Fulop N (2016) Lessons for major system change: centralization of stroke services in two metropolitan areas of England. *Journal of Health Services Research and Policy*; 21:156-65. DOI: 10.1177/1355819615626189.

18. Harris R, Sims S, Hewitt G, Joy M, Brearley S, Cloud G, et al. (2013) Interprofessional teamwork across stroke care pathways: outcomes and patient and carer experience. Final report. NIHR Service Delivery and Organisation programme (Project number 08/1819/219).

19. Hewitt G, Sims S, Harris R (2014) Using realist synthesis to understand the mechanisms of interprofessional teamwork in health and social care. *Journal of Interprofessional Care* **28**(6): 501-506.

20. Sims S, Hewitt G & Harris R (2015) Evidence of collaboration, pooling of resources, learning and role blurring in interprofessional healthcare teams: a realist synthesis. *Journal of Interprofessional Care* **29**(1), 20-25.

21. Sims S, Hewitt G & Harris R (2015) Evidence of a shared purpose, critical reflection, innovation and leadership in interprofessional healthcare teams: a realist synthesis. *Journal of Interprofessional Care* **29**(3): 209-215.

22. King's Fund (2011) *The Future of Leadership and Management in the NHS – No More Heroes*. The King's Fund: London.

23. Naylor R, Dorrell S, Everington S, Lewis R, Morgan G, Serrant L & Stanton E (2015) *Ending the crisis in NHS leadership a plan for renewal.* HSJ Future of NHS Leadership: London.

24. Pawson R, Greenhalgh T, Harvey G, Walshe K (2004). *Realist Synthesis: an introduction*. ESRC Research Methods Programme Methods Paper 2 Accessed via:

For peer review only - http://bmjopen.bmj.com/site/about/guidelines.xhtml

https://www.semanticscholar.org/paper/Realist-synthesis-%3A-an-introduction-Ray-Pawson-Greenhalgh-Harvey/435146e6e6617491ff1c4b32b76e0a534c86d6c7

25. Greenhalgh Y, Wong G, Westhorp G & Pawson R (2011) Protocol - realist and metanarrative evidence synthesis: Evolving Standards (RAMESES). *BMC Medical Research Methodology* **11**:115. DOI: http://www.biomedcentral.com/1471-2288/11/115. 18/01/06

26. Sims S, Leamy M, Davies N, Schnitzler K, Levenson R, Mayer F, Grant R, Brearley S, Gourlay S, Ross F & Harris R (2018) Realist synthesis of intentional rounding in hospital wards: exploring the evidence of what works, for whom, in what circumstances and why. *BMJ Quality and Safety*. DOI: 10.1136/bmjqs-2017-006757

27. Abayneh S, Lempp H, Manthorpe J and Hanlon C (2018). Development of programme theory for integration of service user and caregiver involvement in mental health system strengthening: protocol for realist systematic review. *International Journal of Mental Health Systems* **12**, 41 https://doi.org/10.1186/s13033-018-0220-4

28. Pawson R. (2006). *Evidence-based Policy: A Realist Perspective*. London: Sage

29. Pawson R and Tilley N. (1997). *Realist Evaluation*. London: Sage.

30. Pawson, R. (2006). Digging for nuggets: how 'bad' research can yield 'good' evidence. *International Journal of Sociological Research Methodology* **9**, 127-142.

31. Rycroft-Malone J, McCormack B, Hutchinson AM, DeCorby K, Bucknall TK, Kent B, Schultz A, Snelgrove-Clarke E, Stetler CB, Titler M, Wallin L and Wilson V (2012). Realist synthesis: illustrating the method for implementation research. *Implementation Science* **7**, 33 https://doi.org/10.1186/1748-5908-7-33

32. Wong G, Westhorp G, Manzano A, Greenhalgh J, Jagosh J & Greenhalgh T (2016) RAMESES II reporting standards for realist evaluations. *BMC Medicine* **14**(1):96.

For peer review only - http://bmjopen.bmj.com/site/about/guidelines.xhtml

https://doi.org/10.1186/s12916-016-0643-1

33. Wong G, Westhorp G, Greenhalgh J, Manzano A, Jagosh J & Greenhalgh T (2017) Quality and reporting standards, resources, training materials and information for realist evaluation: the RAMESES II project. *Health Services and Delivery Research*, **5**(28).

For peer review only

For peer review only - http://bmjopen.bmj.com/site/about/guidelines.xhtml

BMJ Open

Understanding key mechanisms of successfully leading integrated team-based services in health and social care: Protocol for a realist synthesis

Journal:	*BMJ Open*
Manuscript ID	bmjopen-2020-038591.R1
Article Type:	Protocol
Date Submitted by the Author:	22-May-2020

Complete List of Authors:	Harris, Ruth; King\'s College London, Florence Nightingale Faculty of Nursing, Midwifery and Palliative Care<br>Fletcher, Simon; Kingston and St Georges University London, Faculty of Health, Social Care and Education<br>Sims, Sarah; King\'s College London, Florence Nightingale Faculty of Nursing, Midwifery and Palliative Care<br>Ross CBE, Fiona; Kingston University and St. George's, University of London, Faculty of Health, Social Care and Education<br>Brearley, Sally; Kingston University and St. George's, University of London, Faculty of Health, Social Care and Education<br>Manthorpe, Jill; King's College London, Health & Social Care Workforce Research Unit

**Primary Subject Heading**:	Health services research
Secondary Subject Heading:	Health policy
Keywords:	Organisation of health services < HEALTH SERVICES ADMINISTRATION & MANAGEMENT, Human resource management < HEALTH SERVICES ADMINISTRATION & MANAGEMENT, Health policy < HEALTH SERVICES ADMINISTRATION & MANAGEMENT, HEALTH SERVICES ADMINISTRATION & MANAGEMENT, Organisational development < HEALTH SERVICES ADMINISTRATION & MANAGEMENT

SCHOLARONE™
Manuscripts

I, the Submitting Author has the right to grant and does grant on behalf of all authors of the Work (as defined in the below author licence), an exclusive licence and/or a non-exclusive licence for contributions from authors who are: i) UK Crown employees; ii) where BMJ has agreed a CC-BY licence shall apply, and/or iii) in accordance with the terms applicable for US Federal Government officers or employees acting as part of their official duties; on a worldwide, perpetual, irrevocable, royalty-free basis to BMJ Publishing Group Ltd ("BMJ") its licensees and where the relevant Journal is co-owned by BMJ to the co-owners of the Journal, to publish the Work in this journal and any other BMJ products and to exploit all rights, as set out in our licence.

The Submitting Author accepts and understands that any supply made under these terms is made by BMJ to the Submitting Author unless you are acting as an employee on behalf of your employer or a postgraduate student of an affiliated institution which is paying any applicable article publishing charge ("APC") for Open Access articles. Where the Submitting Author wishes to make the Work available on an Open Access basis (and intends to pay the relevant APC), the terms of reuse of such Open Access shall be governed by a Creative Commons licence – details of these licences and which Creative Commons licence will apply to this Work are set out in our licence referred to above.

Other than as permitted in any relevant BMJ Author's Self Archiving Policies, I confirm this Work has not been accepted for publication elsewhere, is not being considered for publication elsewhere and does not duplicate material already published. I confirm all authors consent to publication of this Work and authorise the granting of this licence.

For peer review only

Understanding key mechanisms of successfully leading integrated team-based services in health and social care: Protocol for a realist synthesis.

Corresponding author:
Ruth Harris
Florence Nightingale Faculty of Nursing, Midwifery and Palliative Care, King's College London,
James Clerk Maxwell Building, 57 Waterloo Road, London, SE1 8WA, UK
Email: ruth.harris@kcl.ac.uk
Tel: 02078483708
ORCID ID: 0000-0002-4377-5063

Simon Fletcher
Faculty of Health, Social Care and Education,
Kingston University and St George's, University of London, London, UK

Sarah Sims
Florence Nightingale Faculty of Nursing, Midwifery and Palliative Care,
King's College London, London, UK

Fiona Ross CBE
Faculty of Health, Social Care and Education,
Kingston University and St George's, University of London, London, UK

Sally Brearley
Faculty of Health, Social Care and Education,
Kingston University and St George's, University of London, London, UK

Jill Manthorpe
NIHR Policy Research Unit on Health and Social Care Workforce,
King's College London, London, UK
ORCID ID: 0000-0001-9006-1410

Word count: 3269 excluding title page, abstract, references, figures and tables.

For peer review only - http://bmjopen.bmj.com/site/about/guidelines.xhtml

Abstract

Introduction: As systems of health and social care in England move towards more integrated and collaborative models, leaders will need different skills than their predecessors to enable system leadership, building partnerships and working across organisations and sectors. There is little understanding of what the mechanisms for effective leadership across integrated health and social care systems might be, the contexts that influence good leadership, or the nature of the resulting outcomes. This review aims to identify, refine and test programme theories of leadership of integrated team-based services in health and social care, exploring what works, for whom and in what circumstances.

Methods and Analysis: This study uses a realist synthesis approach, following RAMESES guidelines, supported by stakeholder consultation. Stage 1 will develop initial programme theories about leadership of integrated health and social care based on a review of the scientific and grey literature and a stakeholder consultation workshop. Stage 2 will involve focused searching of empirical literature, data extraction and synthesis to refine the initial programme theories and identify relationships between identified contexts, mechanisms and outcomes. A second stakeholder event will guide the focus of the review. Stage 3 will further refine and interrogate the theories testing them against substantive theory on leadership of complex systems and through the experiences and expertise of the stakeholder group.

Ethics and Dissemination: Our study does not require ethics committee approval. This research will contribute to building an in-depth understanding of what aspects of leadership of integrated team-based services work, for whom and in what circumstances. It will identify the professional development needs of leaders and provide recommendations about optimal organisational and inter-organisational structures and processes that support effective leadership in integrated health and social care systems. Findings will be disseminated through peer-reviewed journal publications, conference presentations and formal and informal reports.

Systematic review registration: PROSPERO Number CRD42018119291

For peer review only - http://bmjopen.bmj.com/site/about/guidelines.xhtml

For peer review only

Keywords: Integrated care, leadership, realist synthesis, health and social care

Strengths and limitations of this study

- A realist approach facilitates explanation of the complex nature of leadership of integrated health and social care by making explicit the assumptions of how it is expected to work and investigating the contextual factors that influence this and the subsequent outcomes.
- Consultation with stakeholders with a range of expertise and experience will enable the review to be grounded in the reality of health and social care delivery and address practice and policy challenges.
- The landscape of integrated care in health and social care is rapidly developing and thus the context in which leaders work will change accordingly and so the recommendations of the review may need further consideration.
- Applying a realist evaluation approach can be challenging, and there may be limited evidence to support some elements of the programme theory.

For peer review only - http://bmjopen.bmj.com/site/about/guidelines.xhtml

Background

The Long Term Plan for the UK National Health Service (NHS) and its predecessor strategy, The NHS Five Year Forward View, set out a clear direction for how health and social care services in England must develop to deliver high quality care and treatment in the context of changing patient/user need, increased service delivery pressures and restrained budgets.[1,2] The NHS already works across a wide range of providers and within several sectors and its interdependence between health and social care will certainly increase with new partnerships with local communities, local authorities, and employers being implemented.[2] These new integrated care systems aim to bring together organisations to plan and oversee the implementation of improvements in health and care. It has been argued that current and future integrated care systems must address a range of development needs if they are to be successful, with the development of integrated leaders being vital to this shift.[3]

Leadership is a complex concept. While definitions vary, there is a consensus that a leadership role encompasses the direction of group activities towards shared goals, management of on-going change and support for wider organisational vision, values and objectives.[4,5] Although there are many definitions, some of which seek to differentiate it from management,[6] effective leadership is claimed to be a central element of well-coordinated and safe care.[7-11] Where leadership in health and social care organisations is ineffective (or absent), services fail to meet their aims and patients and service users may be at risk of harm.[12]

Existing research on leadership tends to be based on the notion that leaders provide guidance and support for members of single or uni-professional teams, often based on seniority, authority and deferment.[13,14] However, as the organisation of the NHS and local authority social care commissioning in England becomes more integrated through increasing multi-party, cross-sector and interagency collaboration,[1,15] there is growing

For peer review only - http://bmjopen.bmj.com/site/about/guidelines.xhtml

acknowledgement that leadership is becoming considerably more complex, with specific factors making effective leadership more challenging,[16,17] particularly in negotiating the intricacy and inevitable tensions of expanding integration. Clarity of leadership or conflict in leadership in interprofessional teams has also been found to be highly significant predictors of team performance[18-21] yet there appears to be a tendency for professionals to focus on the support and guidance provided by their own professional leader rather than the leader of the larger interprofessional team.[18,21]

These ongoing and future changes in service delivery mean that health and social care leaders will be less often influencing just one organisation or professional group but instead will be working between several organisations across primary and secondary care, health and social care and publicly funded services, the not-for-profit sector and private businesses. Today's health and social care leaders will therefore need different skills than their predecessors to enable system leadership, building partnerships and working across organisations and sectors.[22,23] However, there is little understanding of what the mechanisms for effective leadership across integrated health and social care systems might be, the contexts that influence good leadership, or the nature of the resulting outcomes.[16] Given this gap in our knowledge and the pressing need to know what might support good leaders in this policy and practice priority area, this realist review focuses on identifying what constitutes good leadership across professional, sector and agency boundaries that seek to promote integrated team-based services across health and social care. The review will undertake an extensive search of the health and social care literature to identify the concepts/theories of leadership to examine what mechanisms work, for whom and in what circumstances. The results from this synthesis, informed by patient, user and carer perspectives, will support the ongoing development and organisation of health/social care, inform leadership development programmes and refine theoretical understandings of leadership to enable future research.

Aims and objectives

This review aims to identify and refine the programme theories of leadership of integrated team-based services in health and social care, exploring what works, for whom and in what circumstances. It will provide practical guidelines for policymakers,

For peer review only - http://bmjopen.bmj.com/site/about/guidelines.xhtml

health and social care leaders, managers and clinicians to help them design work systems and leadership development initiatives to support effective leadership of complex multi-system services. The review started in April 2019.

Objectives:

1. To investigate who are the leaders of integrated team-based services and what activities contribute to their leadership roles and responsibilities.
2. To explore how leaders lead/manage integrated team-based health and social care services that span multiple organisations, agencies and sectors.
3. To develop realist programme theories that explain successful leadership of integrated team-based health and social care services iteratively through stakeholder consultation and evidence review
4. To identify the development needs of the leaders of integrated team-based health and social care services.
5. To provide recommendations about optimal organisational and inter-organisational structures and processes that support effective leadership of the integrated health and social care system.

Methods and analysis

As with all complex social interventions, it can be assumed that leadership *might* work for different stakeholders in various settings in different ways. Therefore, we will conduct a realist review,[24,25] developing and iteratively refining initial programme theories through stakeholder consultation and evidence review. This approach has been successfully used previously by the research team members.[18,26,27] Realist synthesis[28] was developed in order to apply realist methods[29] to the evaluation of evidence. It may be of particular use when exploring a concept as fluid as leadership, as the processes of theoretical reasoning which are required by the approach will enable an interrogation of systems and policy to a depth not possible with more conventional or systematic evidence reviews. Pawson (2006, pp. 73-74)[28] states that: 'There is a need for systematic review to go beyond reportage and summary of an existing state of affairs. The point after all, is to support fresh thinking to revise policy and launch it in new directions'. This suggests an inherently iterative, non-linear approach to encompass multiple literature searches and the constant refinement of

For peer review only - http://bmjopen.bmj.com/site/about/guidelines.xhtml

the evidence-based programme theories. Appraisal of the literature should, in addition, be driven by how well it ‘fits’ into this process rather than being guided by predetermined quality criteria. Utilising this method also allows for the plurality of leadership strategies, the success of which seem often dependant on the unique combination of specific contextual conditions and associated actions.

Our realist review will employ a range of strategies, including searches of relevant research and ‘grey’ literature, health and social care policy documents, and stakeholder and advisory consultation. Given the potential complexity of this review, we plan three separate stakeholder and advisory consultation events during the project to ‘ground’ the review in the lived experiences of leaders of integrated teams and patient/user/carer and public involvement (PPI) members of the review advisory group. We will invite 8-10 individuals with health and social care leadership experiences, realist review expertise and/or health/social care leadership policymaking experience. These individuals will meet with the study advisory group and the research team to elicit “realist theories” on the mechanisms and contexts of leadership. This process is recommended in realist evaluation,[29] as understanding what key stakeholders know about an intervention and their reasoning for or against its implementation are essential to understanding it.

The review will be conducted in three interlinked phases:

Phase 1 *– Development of initial programme theories*

In the first phase, initial programme theories, i.e. purported ideas of ‘what is supposed to happen?’ or ‘how is it supposed to work?’ will be identified and made explicit. This will be undertaken in two ways:

- By examining research, policy and grey literature about leadership of complex integrated teams. Research literature will be identified by electronic searches of databases including Medline, CINAHL, Embase, PsycINFO, Health Management Information Consortium, government and other specialist health and social care websites. The following search strategy will be used in phase 1: “Integrat*” OR “multi-team*” OR “multiteam*” OR “cross-bound*” OR “cross bound*” OR “cross-

For peer review only - http://bmjopen.bmj.com/site/about/guidelines.xhtml

organisation*" OR "cross organisation*" OR "cross-sector*" OR "cross sector*" AND "leader*" (Limiter: English language only, where available). Grey literature relating to policy and organisational-based material will be sought by searching Google Scholar, government and other specialist websites (e.g. Leadership Academy, Skills for Care, King's Fund, Advance HE, The Institute of Healthcare Management, Social Care Online). The research team will independently examine documents to identify any purported mechanisms of leadership (i.e. theories or assumptions about why/how leadership was successful/was expected to work). This will continue until no new mechanisms are identified. Discussion between the research team will generate, through consensus, a combined list of preliminary context-mechanism-outcome configurations of leadership of complex integrated teams/services to be refined throughout the review.

- By consulting with key stakeholders and advisory experts to elicit their theories and assumptions about how leadership of integrated teams works. At the first consultation event, participants will be asked to think about their own knowledge and experience of leadership. For example, health and social care leaders will be asked to comment upon their own personal experiences of leading different teams/services and discuss instances when they felt it worked particularly well or not so well. In realist evaluation, such information is useful for its insight and explanatory nature, which can help identify the contexts and mechanisms which are conducive to the outcome of an intervention.[29] It is likely that the literature identified will be broad and several initial programme theories will be identified. This first stakeholder and advisory consultation event will provide important insights into current (early 2020) priorities around leadership within integrated care that will inform the scope of the literature search and direct the research team towards more pertinent aspects of leadership of complex, integrated teams.

Phase 2 *– Retrieval, review and synthesis of evidence*

In this phase, empirical evidence will be sought and reviewed to refine the programme theories. This phase will be undertaken by two activities:

<u>Evidence review</u>

For peer review only - http://bmjopen.bmj.com/site/about/guidelines.xhtml

Literature searching and screening. First, empirical literature will be identified from electronic searches of databases including Medline, CINAHL, Embase, PsycINFO and Health Management Information Consortium. The following search strategy will be used in phase 2: "Integrat*" OR "multi-team*" OR "multiteam*" OR "cross-bound*" OR "cross bound*" OR "cross-organisation*" OR "cross organisation*" OR "cross-sector*" OR "cross sector*" ***OR "Interorganisation*" OR "Inter-orgnisation*"*** AND "leader*" ***AND "Health"*** (Limiter: English language only, where available)**.** Reference lists of relevant papers will be scanned and citation searches conducted in order to identify further materials. Expert advice about generating relevant search terms will be sought from the University's Library and Information Sciences Specialists and revised as additional key words are generated. Papers and other information that satisfy any of the following criteria will be identified as potentially relevant and will be retrieved for review: describe or evaluate leadership; detail its implementation or development in various settings; address the experience of team leaders, team members, policy makers related to leadership; describe the organisational or political context of leadership; reviews of leadership. Only English language documents will be included in the review of the literature. In line with realist methodology,[30] we will not have specific predetermined inclusion and exclusion criteria based upon research method or quality, but we will report areas of general weakness in evidence and individual study weakness where appropriate. Documents will be selected as relevant based on what they can contribute to theory development and/or refinement. The abstracts of all papers identified by searches will be screened for suitability.

Extraction of key information. All potentially relevant papers will be retrieved and assessed by a member of the research team using a structured data extraction form. It is envisaged that the following information will be recorded for each potentially relevant paper: literature item details – whether descriptive, evaluative or a review paper; health and social care service areas in which leadership is situated; description of leadership activities; any reported outcomes in relation to leadership activities, enabling or inhibiting contexts linked to leadership; clarification and explanation about context-mechanism-outcome configurations. Each of the data extraction forms will be independently examined by at least two members of the research team for inclusion. Data or information

For peer review only - http://bmjopen.bmj.com/site/about/guidelines.xhtml

from each of the included materials will be analysed thematically to provide a comprehensive description of the purported mechanisms of team leadership. Contexts that appear to trigger or inhibit the mechanisms will be identified and outcomes for health and social care staff, teams, organisations and patients/users/carers/family members when the mechanism is present or absent will be noted.

Analysis and synthesis. All extracted information will be analysed and synthesised to identify the relationships between identified mechanisms, contexts and outcomes. This process will draw on the realist review work of Rycroft-Malone et al. (2012)[31] and Wong et al. (2016; 2017)[32,33] which build on Pawson's (2006)[28] earlier work on realist enquiry. In doing so, we will undertake the following: organisation of extracted information into evidence tables representing different bodies of literature; identification of themes across evidence tables in relation to emerging patterns in between mechanisms, contexts and outcomes; and linking the emerging patterns to develop hypotheses. Analysis and synthesis will occur iteratively and are likely to run in parallel, with analysis informing syntheses. We will identify prominent recurrent patterns of context and outcome configurations and seek to explain how these occurred.

Stakeholder and advisory consultation

At the second event, participants will be asked to provide advice to the research team with the volume of data generated from the searches. If available theories are limited within the literature, the consultation event could generate additional theories that were not previously identified. If the number of theories is unwieldy, the stakeholder event will enable participants to advise the research team on which should be selected for further investigation.

Phase 3 *– Testing and refining of the initial programme theory*

In this phase planned for 2020, further refinement and testing of the programme theories will be undertaken by juxtaposing, adjudicating, reconciling, consolidating and situating the evidence analysed in phase 2.[28] This will generate a revised programme theory. This final phase will consist of the following activities:

For peer review only - http://bmjopen.bmj.com/site/about/guidelines.xhtml

- The research team will interrogate each explanatory inference identified in Phase 2 to elicit how each primary study supports, weakens, modifies and refocuses the initial programme theories and how particular mechanisms produce outcomes, within specific contexts. Comparisons between contexts and different types of health and social care services will be sought to test the refined programme theories and draw out patterns of demi-regularity. Furthermore, the refined programme theories will be tested against substantive theory on leadership of complex systems.
- At the final consultation event (mid 2020), interpretations from Phase 3 of the review will be tested through the experiences and expertise of the stakeholder/advisory group. Recommendations will be sought about what leadership mechanisms may be of most benefit and what contextual factors might support their success. Furthermore, participants will be asked to consider how best to present study findings and outputs to be useful to the NHS and social care system.

Ethics and dissemination

As this is a review and synthesis of literature no formal ethical approval is needed. However, the team will apply good practices of research governance and conduct. We will follow RAMESES Guidelines[32,33] when reporting the findings from this review. It is anticipated that the final report containing synthesised review findings will identify the underlying mechanisms of leadership of integrated team-based services, and explain how these produce their effects, as well as highlight the main contextual factors that impact success or failure. Recommendations can then be made as to how employers can best target or develop integrated team leadership development for specific groups in various settings.

We will draw on the networks and expertise of the research team, advisory group and collaborators to disseminate the research outputs widely and appropriately. Key audiences are expected to include:

- Health and social care staff, managers and leaders together with clinical and human resource directors who have responsibility for the provision of health and social care in provider organisations, local authorities, voluntary and private sector as well as NHS acute, mental health and community Trusts
- Managers and directors of networks, e.g. in England these include Academic Health Sciences Networks and the NIHR Applied Research Collaborations (ARCs) who have

For peer review only - http://bmjopen.bmj.com/site/about/guidelines.xhtml

responsibility for applied research, knowledge exchange and promoting innovation through pathways of care across a health and social care system

- Health and social care policymakers, nationally and internationally
- Health Education England, Royal Colleges and other leadership groups (e.g. NHS Leadership Academy, Skills for Care, Local Government Association, Care England, National Care Forum, King's Fund, Advance HE, The Institute of Healthcare Management, NHS Improvement), who are influential in policy concerning leadership in different contexts
- National patient/ service user and carer organisations.

Patient and public involvement

One member of the research team (SB) has a great deal of experience of patient and public involvement, for example, she is currently a Lay Member on the Interventional Procedures Advisory Committee at the National Institute for Health and Care Excellence (NICE) and has chaired many service user and carer research advisory groups at a national and local level. She has an established reputation as a co-applicant on NIHR grants and taken an active role in conduct and delivery of research, including writing for publication. As a co-applicant on this grant, she was involved in writing the protocol, developing the review questions and methods, and takes a full part in research team meetings and in particular planning the stakeholder and advisory group meetings and follow up. Three members of the public who have expertise in providing patient/service user expertise in health and social care services are part of the independent study advisory group and actively involved in the development and refinement of programme theories. Their expertise and perspectives will also be sought to develop recommendations and contribute to the study dissemination strategy.

Discussion

This realist review will enable a novel theoretical and strategically practical response to the relative lack of knowledge around the leadership of integrated health and social care services. It aligns with England's current priorities in health and social care and will provide new insights about how the leadership needs of these services are changing.

For peer review only - http://bmjopen.bmj.com/site/about/guidelines.xhtml

The need for integrated care teams and systems is an increasing priority in response to fragmented and un-coordinated care delivery. Ageing and diverse populations, increasing inequalities, associated co-morbidities and a continual increase in long-term conditions require the collaborative interdependence of health and care providers to reduce harm and improve well-being. However, the simplicity of this statement belies the complexity of the landscape. Effective leadership is needed in order to mediate the shared objective of improving health and well-being outcomes and the altogether more difficult management of workforce interaction and the associated disruption of organisational change.

Declarations

Ethics approval and consent to participate – Not applicable

Consent for publication - Not applicable

Availability of data and material - The datasets generated and/or analysed during the current study will be available from the corresponding author on reasonable request.

Competing interests – The authors declare that they have no competing interests.

Funding - This project is funded by the National Institute for Health Research (NIHR) Health Service and Delivery Research (HS&DR) (project reference 18/01/06). The views expressed are those of the authors and not necessarily those of the NIHR or the Department of Health and Social Care.

Acknowledgements – This project was originally conceived and developed with the late Professor Scott Reeves, who passed away unexpectedly on May 3rd 2018 shortly after the funding application was submitted. His original ideas and considerable expertise in interprofessional healthcare research were instrumental in the formation of this study and the research team dedicate this project to his memory.

Contributors - RH conceived the study and drafted the protocol. SF, FR, SS, SB and JM contributed to refining the study objectives, design and methodology. SF and RH drafted the

For peer review only - http://bmjopen.bmj.com/site/about/guidelines.xhtml

paper and FR, JM, SS and SB have reviewed and provided comments to improve the paper. All authors have read and approved the final version.

For peer review only

References

1. NHS England (2014). *The NHS Five-year forward view.* Available at: https://www.england.nhs.uk/publication/nhs-five-year-forward-view/

2. NHS England (2019). *The NHS Long Term Plan*. Available at: https://www.longtermplan.nhs.uk/

3. Ham C (2018). *A progress report on integrated care systems.* Available online at: https://www.kingsfund.org.uk/blog/2018/03/progress-report-integrated-care-systems

4. Dopson S & Annabelle M (2003). Leading Health Care Organizations. Palgrave Macmillan, Basingstoke.

5. Dopson S, Ferlie E, McGivern G, Fischer M, Ledger J, Behrens S & Wilson S (2016). *The impact of leadership and leadership development in higher education: A review of the*

For peer review only - http://bmjopen.bmj.com/site/about/guidelines.xhtml

literature and evidence. Leadership Foundation for Higher Education, London.

6. Iliffe S & Manthorpe J (2017). Service integration through medical leadership in England's NHS. *Journal of Integrated Care* **26**, 77-86.

7. NHS Leadership Academy (2011). *Clinical Leadership Competency Framework.* Institute for Innovation and Improvement: Coventry.

8. Drucker P. (2002). *Managing in the Next Society.* New York: Truman Talley Books, St. Martin's Griffin.

9. NIHR (2013). *New Evidence on Management and Leadership*. National Institute for Health Research, Southampton.

10. Ahmed N, Ahmed F, Anis H, Carr P, Gauher S & Rahman F (2015). An *NHS leadership team for the future.* Reform Research Trust: London.

11. Smith T, Fowler-Davis S, Nancarrow S, Ariss S & Enderby P (2018). *Leadership in interprofessional health and social care teams: A literature review.* Leadership in Health Services. Accessed via: https://doi.org/10.1108/LHS-06-2016-0026.

12. Francis R. (2013). *Report of the Mid Staffordshire NHS Foundation Trust Public Inquiry, (Chair Robert Francis QC)*. London: The Stationery Office.

13. Reeves S, MacMillan K, van Soeren M (2010). Leadership within interprofessional health and social care teams: a socio-historical overview of some key trials and tribulations. *Journal of Nursing Management* **18**, 258-264.

14. Borkowski N (2015). *Organizational behavior in health care.* Jones & Barlett Learning: Burlington MA.

For peer review only - http://bmjopen.bmj.com/site/about/guidelines.xhtml

15. Ham C & Murray R (2015). *Implementing the five year forward view: aligning policy to the plan.* Available online at: www.kingsfund.org.uk/publications/implementing-nhs-five-yearforward-view

16. Fillingham D & Weir B (2014). *System leadership. Lessons and learning from AQuA's Integrated Care Discovery Communities.* King's Fund: London.

17. Turner S, Ramsay A, Perry C, Boaden R, McKevitt C, Morris S, Pursani N, Rudd A, Tyrrell P, Wolfe C & Fulop N (2016) Lessons for major system change: centralization of stroke services in two metropolitan areas of England. *Journal of Health Services Research and Policy*; 21:156-65. DOI: 10.1177/1355819615626189.

18. Harris R, Sims S, Hewitt G, Joy M, Brearley S, Cloud G, et al. (2013) Interprofessional teamwork across stroke care pathways: outcomes and patient and carer experience. Final report. NIHR Service Delivery and Organisation programme (Project number 08/1819/219).

19. Hewitt G, Sims S, Harris R (2014) Using realist synthesis to understand the mechanisms of interprofessional teamwork in health and social care. *Journal of Interprofessional Care* **28**(6): 501-506.

20. Sims S, Hewitt G & Harris R (2015) Evidence of collaboration, pooling of resources, learning and role blurring in interprofessional healthcare teams: a realist synthesis. *Journal of Interprofessional Care* **29**(1), 20-25.

21. Sims S, Hewitt G & Harris R (2015) Evidence of a shared purpose, critical reflection, innovation and leadership in interprofessional healthcare teams: a realist synthesis. *Journal of Interprofessional Care* **29**(3): 209-215.

22. King's Fund (2011) *The Future of Leadership and Management in the NHS – No More Heroes*. The King's Fund: London.

23. Naylor R, Dorrell S, Everington S, Lewis R, Morgan G, Serrant L & Stanton E (2015) *Ending*

For peer review only - http://bmjopen.bmj.com/site/about/guidelines.xhtml

the crisis in NHS leadership a plan for renewal. HSJ Future of NHS Leadership: London.

24. Pawson R, Greenhalgh T, Harvey G, Walshe K (2004). *Realist Synthesis: an introduction*. ESRC Research Methods Programme Methods Paper 2 Accessed via: https://www.semanticscholar.org/paper/Realist-synthesis-%3A-an-introduction-Ray-Pawson-Greenhalgh-Harvey/435146e6e6617491ff1c4b32b76e0a534c86d6c7

25. Greenhalgh Y, Wong G, Westhorp G & Pawson R (2011) Protocol - realist and metanarrative evidence synthesis: Evolving Standards (RAMESES). *BMC Medical Research Methodology* **11**:115. DOI: http://www.biomedcentral.com/1471-2288/11/115. 18/01/06

26. Sims S, Leamy M, Davies N, Schnitzler K, Levenson R, Mayer F, Grant R, Brearley S, Gourlay S, Ross F & Harris R (2018) Realist synthesis of intentional rounding in hospital wards: exploring the evidence of what works, for whom, in what circumstances and why. *BMJ Quality and Safety*. DOI: 10.1136/bmjqs-2017-006757

27. Abayneh S, Lempp H, Manthorpe J and Hanlon C (2018). Development of programme theory for integration of service user and caregiver involvement in mental health system strengthening: protocol for realist systematic review. *International Journal of Mental Health Systems* **12**, 41 https://doi.org/10.1186/s13033-018-0220-4

28. Pawson R. (2006). *Evidence-based Policy: A Realist Perspective*. London: Sage

29. Pawson R and Tilley N. (1997). *Realist Evaluation*. London: Sage.

30. Pawson, R. (2006). Digging for nuggets: how 'bad' research can yield 'good' evidence. *International Journal of Sociological Research Methodology* **9**, 127-142.

31. Rycroft-Malone J, McCormack B, Hutchinson AM, DeCorby K, Bucknall TK, Kent B, Schultz A, Snelgrove-Clarke E, Stetler CB, Titler M, Wallin L and Wilson V (2012). Realist synthesis:

For peer review only - http://bmjopen.bmj.com/site/about/guidelines.xhtml

illustrating the method for implementation research. *Implementation Science* **7**, 33 https://doi.org/10.1186/1748-5908-7-33

32. Wong G, Westhorp G, Manzano A, Greenhalgh J, Jagosh J & Greenhalgh T (2016) RAMESES II reporting standards for realist evaluations. *BMC Medicine* **14**(1):96. https://doi.org/10.1186/s12916-016-0643-1

33. Wong G, Westhorp G, Greenhalgh J, Manzano A, Jagosh J & Greenhalgh T (2017) Quality and reporting standards, resources, training materials and information for realist evaluation: the RAMESES II project. *Health Services and Delivery Research*, **5**(28).

For peer review only

For peer review only - http://bmjopen.bmj.com/site/about/guidelines.xhtml
