# Supplementary material for: Understanding key mechanisms of successfully leading integrated team-based services in health and social care: protocol for a realist synthesis
Source: BMJ Open. 2020 Jul 9;10(7):e038591. doi: 10.1136/bmjopen-2020-038591 (PMC7351270; doi:10.1136/bmjopen-2020-038591)
Supplement: Reviewer comments [file bmjopen-2020-038591.reviewer_comments.pdf]

PEER REVIEW HISTORY

BMJ Open publishes all reviews undertaken for accepted manuscripts. Reviewers are asked to complete a checklist review form (**http://bmjopen.bmj.com/site/about/resources/checklist.pdf**) and are provided with free text boxes to elaborate on their assessment. These free text comments are reproduced below.

ARTICLE DETAILS

**TITLE (PROVISIONAL)**	Understanding key mechanisms of successfully leading integrated team-based services in health and social care: Protocol for a realist synthesis
**AUTHORS**	Harris, Ruth; Fletcher, Simon; Sims, Sarah; Ross CBE, Fiona; Brearley, Sally; Manthorpe, Jill

This article was not externally reviewed at BMJ Open. Protocols that have been independently assessed prior to submission to BMJ Open are usually fast-tracked to publication on the grounds that further substantial changes will not be possible. This independent assessment will usually be external, independent review for both a substantial grant award from a non-commercial or government funder and ethics approval.
